# The Computerized Table Setting Test for Detecting Unilateral Neglect

**DOI:** 10.1371/journal.pone.0147030

**Published:** 2016-01-15

**Authors:** Seok Jong Chung, Eunjeong Park, Byoung Seok Ye, Hye Sun Lee, Hyuk-Jae Chang, Dongbeom Song, Young Dae Kim, Ji Hoe Heo, Hyo Suk Nam

**Affiliations:** 1 Department of Neurology, Yonsei University College of Medicine, Seoul, Korea; 2 Wireless Health Institute, University of California Los Angeles, Los Angeles, United States of America; 3 Department of Cardiology, Yonsei University College of Medicine, Seoul, Korea; 4 Department of Biostatistics, Yonsei University College of Medicine, Seoul, Korea; University Zurich, SWITZERLAND

## Abstract

**Background:**

Patients with unilateral neglect fail to respond normally to stimuli on the left side. To facilitate the evaluation of unilateral spatial neglect, we developed a new application that runs on a tablet device and investigated its feasibility in stroke patients.

**Methods:**

We made the computerized table setting test (CTST) to run on the tablet computer. Forty acute ischemic stroke patients (20 patients with right hemispheric infarction with neglect, 10 patients with right hemispheric infarction without neglect, and 10 patients with left hemispheric infarction) and 10 healthy controls were prospectively enrolled to validate the CTST. The test requires subjects to set a table by dragging 12 dishes located below the table on the tablet screen. The horizontal deviation of the 12 dishes from the midline of the table, the selection tendency measured by the sequence of the dish selection, and the elapsed time for table setting were calculated automatically.

**Results:**

Parameters measured by the CTST were correlated with the results of conventional neglect tests. The horizontal deviation was significantly higher in patients with right hemispheric infarction with neglect compared with the other groups. The selection tendency and elapsed time also were significantly different in patients with right hemispheric infarction with neglect compared with the left hemispheric infarction and control groups, but were similar to those with right hemispheric infarction without neglect.

**Conclusions:**

The CTST is feasible to administer and comparable with conventional neglect tests. This new application may be useful for the initial diagnosis and follow-up of neglect patients.

## Introduction

Neglect syndrome refers to the failure of attending or responding to stimuli in the contralesional space. Neglect is classified as unilateral spatial neglect, extinction to double simultaneous stimulation, motor neglect, sensory neglect, personal neglect, and anosognosia for hemiplegia [1]. Neglect is more frequently associated with damage to the right cerebral hemisphere and is a poor prognostic factor for functional recovery after stroke [2].

Many tests have been developed to assess neglect, and most of them are the paper-and pencil tests [3–5]. An ideal neglect test is reasonably sensitive and simple to perform, provides objective data to compare the degree of neglect, and is easy to interpret [5]. Although the existing conventional neglect tests are useful, it is not easy for a single test to satisfy these ideal requirements. In addition, a test that is relevant to activities of daily living and able to cover various features of neglect might be more useful than conventional neglect tests [6].

Recently, mobile devices have been used as supplementary tools for neurological examination and clinical decision-making [7–9]. Among existing mobile devices, tablets provide rich multi-touch user interfaces and a large screen that allows patients to perform behaviour tasks easily [10,11]. Tablets also provide sufficient computation power for measurement, calculation, data storage, and real-time analysis. In this regard, a tool for detecting and quantifying neglect that runs on a tablet device would be helpful. Therefore, we developed a computerized neglect test that runs on a tablet device, and evaluated its usefulness in patients with ischemic stroke.

## Patients and Methods

### Subjects

Subjects for this study were prospectively enrolled to validate the computerized table setting test (CTST). A neurological examination, including the cortical function test, cranial nerve function test, motor and sensory examination, cerebellar function, language, and neglect tests was performed in each patient. The presence of neglect, including visual neglect, auditory neglect, tactile extinction, asomatognosia, and anosognosia, was tested in all patients. In this study, clinical neglect was defined when the patient exhibited at least one of these neglect signs during a bedside neurological examination. Exclusion criteria were 1) lacunar infarction, brainstem/cerebellar infarction, or bilateral hemispheric infarction; 2) history of previous stroke; 3) inability to perform the neglect tests (e.g. altered consciousness, poor cooperation, aphasia, or severe medical or surgical illness); 4) no diffusion-weighted MRI (DWI); and 5) bilateral hemispheric lesions on DWI.

Study subjects were divided into four groups according to the presence of neglect and the location of ischemic lesions on DWI: right hemispheric infarction (RHI) with neglect, RHI without neglect, left hemispheric infarction (LHI), and healthy controls without a history of neurological disease. The study was approved by the Institutional Review Board of Severance Hospital, Yonsei University Health System. Written informed consent was obtained from all subjects participating in this study.

### Development of the computerized table setting test

We developed the CTST to run on the iPad (Apple Inc., Cupertino, CA, USA). The CTST was coded with the software development toolkit (iOS SDK 4.0, Apple Inc., Cupertino, CA, USA). During the CTST, a virtual table with 12 dishes is shown in the middle of screen. Subjects are instructed to set the table by dragging individual dishes, which are located below the table, to the top of the table. Once subjects have set the table with all 12 dishes, three parameters are instantly calculated: the horizontal deviation from the midline, the selection tendency for dishes on the right, and the elapsed time to set the table.

**Fig 1:**
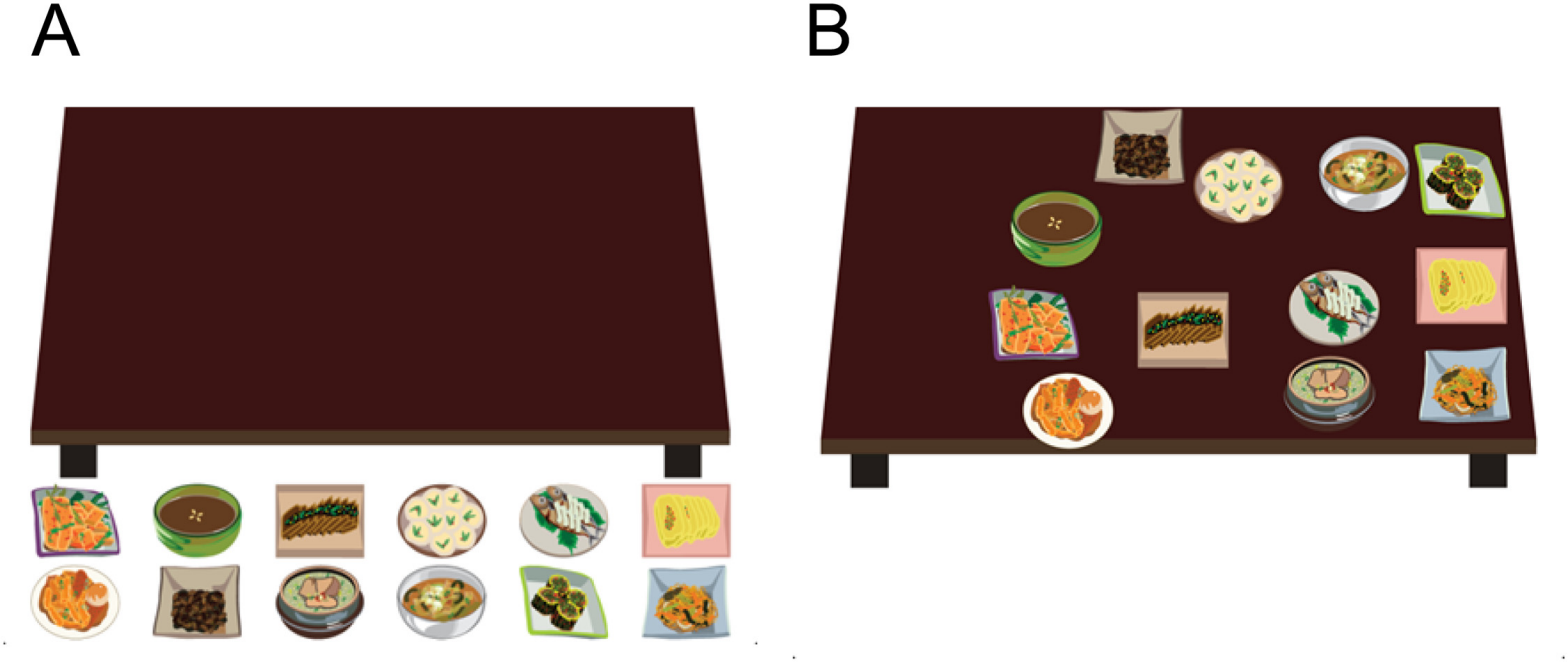
The computerized table setting test (CTST) for the iPad. In the CTST, twelve dishes are located below the virtual table. The subject was instructed to set the table by dragging dishes to the tabletop (A). After setting the table, the horizontal deviation, selection tendency, and elapsed time are automatically measured and recorded (B).

The horizontal deviation of all 12 dishes was determined in real-time by calculating the mean distance between the middle of the screen and the centre of each dish. A positive value is indicative of a rightward deviation from the midline and a negative value is indicative of a leftward deviation. The CTST was designed to measure a tendency to initiate the task from the dishes on the right [12]. To measure the selection tendency, a sequence of the first 3 dish selection attempts was recorded and weighted. The weighting method that we employed was a numerical scale in which 6 points was applied to the most right-sided dishes and 1 point was applied to the most left-sided dishes. Therefore, a higher value is indicative of a more right-sided selection tendency and a lower value is indicative of a more left-sided selection tendency. For example, if the subject selected a sequence of three right-sided dishes (two of the first right column dishes and one of the second right column dishes), the selection tendency was calculated as the mean of the attempts [i.e. (6 + 6 + 5) / 3 = 5.7]. The elapsed time for the CTST also was calculated and recorded in real-time (Fig 1).

### Conventional neglect tests

To investigate the feasibility of the CTST, we compared commonly performed the paper-and-pen based neglect tests. The line bisection test and the star cancellation test were conducted using previously described methods [13,14].

#### The line bisection test

Each subject was instructed to mark the midpoint of a given horizontal line (242 mm long and 1.5 mm thick) on an A4 sheet (297 mm x 210 mm) with a black felt-tip pen. The midline of the paper was aligned with the mid-sagittal plane of the patient. The deviations from the midpoint (mm) were measured with a positive value for right-sided deviations and a negative value for left-sided deviations. These deviations were transformed to a 10-point scale by dividing the mean deviations in five attempts by half the length of the line (121 mm) and multiplying this value by 10. Therefore, the score of the line bisection test ranged from -10 to +10 [13].

#### The star cancellation test

Each patient was asked to cross a total of 56 small stars that were randomly arranged on an A4 sheet. Twenty-seven small stars on each half of the page were analysed; two small stars in the centre of the page were not counted [15]. The laterality index (LI) and severity index (SI) were calculated according to the following formula: LI = (marked stars on the right—marked stars on the left)/(marked stars on the right + marked stars on the left); SI = (unmarked stars)/(total number of stars). The star cancellation score was calculated by multiplying LI and SI and then multiplying this value by 10. The score of the star cancellation test ranged from -10 to +10. A score of +10 was given when the patient marked stars on the right side only and a score of -10 was given when the patient marked stars on the left side only. Thus, a higher positive score represents more deviations to the right (consistent with LI) and less general attention (consistent with SI) [13,16,17].

#### Total neglect score of conventional tests

A total neglect score assessed the overall performance on the conventional neglect tests. The total neglect score was defined as the sum of the line bisection score and the star cancellation score and ranged from -20 to +20.

### Sensitivity of individual neglect tests

We calculated the sensitivity of the CTST and the conventional neglect tests. Analyses were performed in RHI with neglect group. To calculate the sensitivity, we used the scores of healthy subjects. According to previous studies, a cut-off score was defined as a score exceeding the mean plus 2 SD of healthy subjects’ scores when the data were normally distributed, whereas the lowest performance of healthy subjects minus one when the data showed non-normal distribution [13,15]. For the all parameters of the CTST and the line bisection test, the scores of healthy subjects showed normal distribution. For the star cancellation tests, all but four healthy subjects marked all the stars on the paper and the dataset showed non-normal distribution.

### Infarction volume measurement

All patients underwent MRI study using a 3.0T MRI system (Achieva 3.0T; Philips Medical Systems, Best, The Netherlands or MAGNETOM 3.0T Trio; Siemens, Germany). We measured the infarction volumes of patients with RHI (20 RHI with neglect and 10 RHI without neglect) using a commercially available software (Xelis; INFINITT Healthcare, Seoul, Korea) [18]. A rater who was blinded to the patient’s condition measured the infarction volume to minimize bias.

### Follow-up tests for the patients with neglect

Among the 20 patients of RHI with neglect, follow-up tests were performed in 10 patients. Other 10 patients were not included because one died, three declined participation, and six of them were lost to follow-up. The National Institutes of Health Stroke Scale (NIHSS) scores and presence of clinical neglect were also evaluated and compared with baseline results.

### Statistical analysis

Statistical analyses were performed with SPSS version 18.0 (SPSS Inc., Chicago, IL, USA), SAS version 9.2 (SAS Institute, Inc., Cary, NC, USA), and R package version 2.9.2 (http://www.R-project.org). Normal distributions of the dataset were tested using Kolmogorov-Smirnov test.

A one-way analysis of variance (ANOVA) with a Bonferroni correction for multiple comparisons was used to compare the demographic characteristics and degree of neglect among the four groups. The Pearson’s correlation coefficient was calculated to assess the relationship between neglect tests. The Student’s *t*-test was used to compare the lesion volume in patients with RHI according to the presence of neglect, and Pearson’s correlation coefficient was used to assess the relationship between the lesion volume and degree of neglect as measured by the CTST in the 30 RHI patients. Paired t-test was used to compare the repeatedly measured data. A univariate analysis tested the association between the presence of clinical neglect and parameters obtained from the CTST. Multivariate logistic regression analyses were performed and included independent factors that were not multi-collinear. We developed a nomogram to predict the presence of neglect [19,20]. The performance of the nomogram was quantified with respect to discrimination and calibration. Using the area under the curve (AUC), the discrimination power whether nomogram can correctly ordered the ranking of probability was quantified. The ability of the nomogram to discriminate the probability of a clinical diagnosis was quantified using calibration curves. A two-tailed p < 0.05 was considered statistically significant.

## Results

### Subject characteristics

Forty patients (23 men and 17 women) with acute ischemic stroke were enrolled. The mean age was 66.3 ± 11.6 years. The CTST and conventional neglect tests were performed at a median of 5 days [interquartile range (IQR) 3–8 days] after acute ischemic stroke. The groups consisted of RHI with neglect (n = 20), RHI without neglect (n = 10), LHI (n = 10), and healthy subjects (n = 10) (Table 1).

**Table 1:** Demographic characteristics and the results of the CTST and the conventional tests.

	RHI with neglect (n = 20)	RHI without Neglect (n = 10)	LHI (n = 10)	Control (n = 10)	P
Age	69.2 ± 10.2	65.5 ± 13.0	61.1 ± 12.0	46.0 ± 15.2	< 0.001
Sex (men, %)	10 (50%)	2 (20%)	5 (50%)	5 (50%)	0.449
NIHSS score	7.5 ± 4.3	1.4 ± 1.2	1.9 ± 2.8	-	< 0.001
Onset to tests (days)	8.0 ± 6.3	4.0 ± 1.8	6.8 ± 6.6	-	0.202
**CTST**					
Horizontal deviation (mm)	16.01 ± 17.97	1.68 ± 6.99	-0.29 ± 5.43	-2.92 ± 6.78	< 0.001
Selection tendency	5.01 ± 0.87	4.02 ± 1.57	2.17 ± 0.65	2.22 ± 0.93	< 0.001
Elapsed time (sec)	74.75 ± 52.41	45.20 ± 25.38	35.60 ± 18.03	28.90 ± 10.37	0.006
**Conventional tests**					
Line bisection (mm)	1.58 ± 1.88	0.16 ± 1.01	-0.54 ± 1.26	-0.15 ± 0.35	0.001
Star cancellation	3.67 ± 4.25	0.10 ± 0.33	0.05 ± 0.20	-0.01 ± 0.03	0.001
Total neglect score	4.90 ± 5.20	0.29 ± 1.14	-0.49 ± 1.15	-0.15 ± 0.37	< 0.001

The values are expressed as the mean ± standard deviation or number (percentage).

CTST = the computerized table setting test; RHI = right hemispheric infarction; LHI = left hemispheric infarction; NIHSS, National Institutes of Health Stroke Scale.

### Correlation between the CTST and conventional neglect tests

Correlation analysis revealed that all three parameters of the CTST were significantly correlated with the total neglect score (horizontal deviation, r = 0.699, p < 0.001; selection tendency, r = 0.565, p < 0.001; and elapsed time, r = 0.379, p = 0.007). Among the parameters obtained from the CTST, a significant correlation was observed between the horizontal deviation and selection tendency (r = 0.485, p < 0.001) and between the selection tendency and elapsed time (r = 0.427, p = 0.002). Elapsed time was not correlated with horizontal deviation of the CTST and the line bisection test of conventional test (Table 2).

**Table 2:** Correlation analysis between the CTST and the conventional tests.

	CTST		
	Horizontal deviation	Selection tendency	Elapsed time
**Conventional tests**			
Line bisection	0.694 (< 0.001)	0.612 (< 0.001)	0.151 (0.297)
Star cancellation	0.575 (< 0.001)	0.468 (0.001)	0.463 (0.001)
Total neglect score	0.699 (< 0.001)	0.565 (< 0.001)	0.379 (0.007)
**CTST**			
Horizontal deviation	1.000		
Selection tendency	0.485 (< 0.001)	1.000	
Elapsed time	0.268 (0.06)	0.427 (0.002)	1.000

The values are expressed as the correlation coefficient (p-value).

CTST = the computerized table setting test.

### Comparison of neglect test results between groups

**Fig 2:**
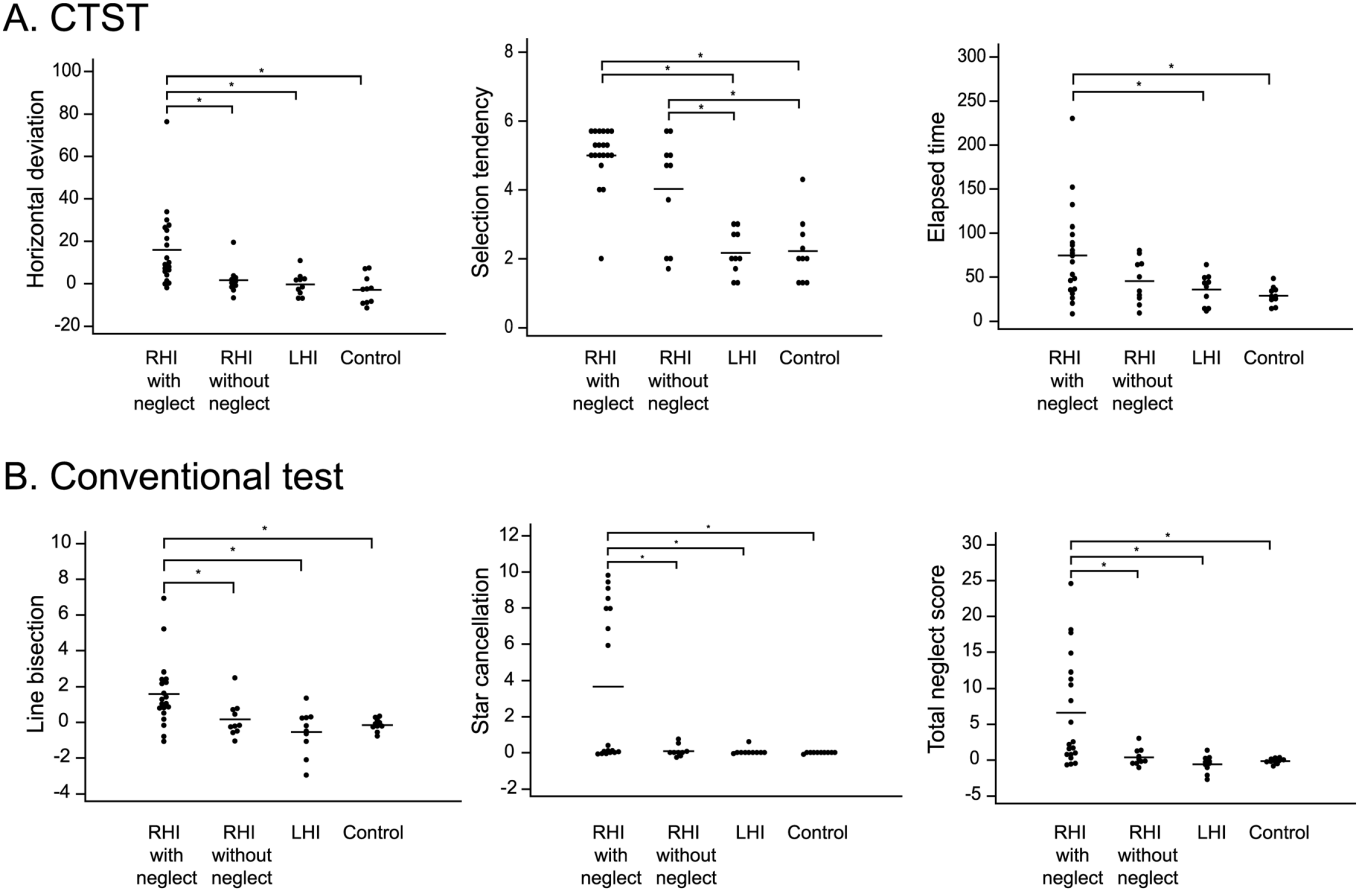
Comparison of the CTST parameters and neglect scores assessed by conventional neglect tests across groups. The horizontal deviation of RHI with neglect group was different from that of RHI without neglect group (p = 0.004), LHI group (p = 0.001), and control group (p = 0.003). The selection tendency and the elapsed time were not different between RHI with neglect group and RHI without neglect group. In contrast, the selection tendency was significantly different in comparison between RHI without neglect group and either LHI group or control group. The elapsed time was significantly different in RHI with neglect group compared with LHI group and healthy control group (A). In the conventional tests, the scores were different between RHI with neglect group and other groups (B). RHI = right hemispheric infarction; LHI = left hemispheric infarction.

All three parameters of the CTST were significantly different between groups (horizontal deviation, p < 0.001; selection tendency, p < 0.001; and elapsed time, p = 0.006) (Table 1). Post-hoc analyses revealed that the horizontal deviation of the CTST can differentiate between RHI with neglect group and RHI without neglect group (p = 0.004). The horizontal deviation of RHI with neglect group was different from that of LHI group (p = 0.001) and control group (p = 0.003). The selection tendency and the elapsed time were not different between RHI with neglect group and RHI without neglect group. In contrast, the selection tendency was significantly different in comparison between RHI without neglect group and either LHI group (p = 0.005) or control group (p = 0.007). The elapsed time was significantly different in RHI with neglect group compared with LHI group (p = 0.031) and healthy control group (p = 0.001) (S1 Table and Fig 2A). In the conventional tests, the scores were significantly different between groups. Post-hoc analyses revealed that the scores of conventional neglect tests were different between RHI with neglect group and other groups. Comparison among RHI without neglect, LHI, and control groups were not different (S1 Table and Fig 2B).

### Sensitivity of individual neglect tests

Sensitivities of the CTST were 45% of the horizontal deviation, 85% of the selection tendency, and 60% of the elapsed time. Because the CTST can simultaneously get these three parameters as soon as the completion of table setting, we calculate the overall sensitivity of the CTST. The overall sensitivity of the CTST was 95% (the overall sensitivity was defined when the patient had a higher score than a cut-off in at least one of parameters in the CTST). Among conventional tests, the sensitivity of the line bisection test was 75%. That of the star cancellation test was 94.4% (17 out of 18).

### Association between infarction volume and the CTST

Significant correlations were observed between the infarction volume and the CTST parameters. A larger infarction volume was associated with a greater horizontal deviation (r = 0.375, p = 0.041) and a longer elapsed time (r = 0.628, p < 0.001), whereas the selection tendency was not correlated with the infarction volume (r = 0.139, p = 0.465). Among the RHI patients, the RHI with neglect group had larger infarction volumes than the RHI without neglect group (40.77 ± 41.52 cm^3^ vs. 9.43 ± 13.60 cm^3^, p = 0.005) (S2 Table).

### Follow-up tests in the patients with neglect

Follow-up neglect tests were performed in 10 patients of RHI with neglect group. The median time interval from initial evaluation to follow-up tests was 256 days (IQR 187–375). The NIHSS scores were significantly improved from 6.1 ± 4.4 to 3.1 ± 4.0 (p < 0.001) during follow-up. The horizontal deviation (from 10.78 ± 10.83 to 1.30 ± 6.04, p = 0.026) and the selection tendency (from 5.11 ± 0.53 to 3.09 ± 1.59, p = 0.002) in the CTST were significantly improved at the follow-up tests. For the conventional tests, the score of the line bisection test and the total neglect score were improved. Whereas, the elapsed time in the CTST (from 64.10 ± 35.13 to 39.50 ± 29.78, p = 0.054) and the score of the star cancellation test were not significantly improved at follow-up evaluations (Table 3).

**Table 3:** Baseline and follow-up findings in patients with right hemispheric infarction with neglect.

	Baseline (n = 10)	Follow-up (n = 10)	P
Onset to tests (day)	8.9 ± 8.0	292.3 ± 111.4	< 0.001
NIHSS score	6.1 ± 4.4	3.1 ± 4.0	< 0.001
Clinical neglects	10 (100%)	3 (30%)	
**CTST**			
Horizontal deviation	10.78 ± 10.83	1.30 ± 6.04	0.026
Selection tendency	5.11 ± 0.53	3.09 ± 1.59	0.002
Elapsed time (sec)	64.10 ± 35.13	39.50 ± 29.78	0.054
**Conventional tests**			
Line bisection	1.67 ± 1.51	0.14 ± 0.49	0.004
Star cancellation	3.05 ± 4.30	0.45 ± 0.80	0.083
Total neglect score	4.14 ± 4.28	0.60 ± 1.03	0.008

The values are expressed as mean ± standard deviation or number (percentage). Abbreviations: NIHSS, National Institutes of Health Stroke Scale; CTST, computerized table setting test.

### Constructing a nomogram

Univariate logistic analysis revealed that clinical neglect was associated with age (p = 0.01) and a right hemispheric lesion (p < 0.001). On the CTST, all three parameters were associated with the presence of clinical neglect. Among the conventional tests, the line bisection test and the total neglect score were associated with clinical neglect. In the multivariate logistic regression analysis, including the horizontal deviation and the selection tendency of the CTST was the most powerful model to predict clinical neglect (Table 4). An equation to predict the probability of clinical neglect was determined from the multivariate logistic regression. The probability of neglect is calculated as 1/(1+exp(-A)), where A = - 4.9061 + 0.1589 × (the horizontal deviation) + 0.9446 × (the selection tendency). This equation was embedded in the CTST program, and the probability of clinical neglect was displayed immediately after the completion of the test. We constructed a nomogram according to these two parameters. In the nomogram, each point of the horizontal deviation and selection tendency was calculated. A sum of each point was regarded as a total point. A corresponding probability of clinical neglect can be estimated from each total point. An internal validation of the nomogram revealed that the ability of the nomogram to discriminate the presence of clinical neglect was excellent [AUC of 0.937 (95% CI, 0.873 to 1.000)]. In terms of calibration, the predicted probabilities from the equation and the actual probability of the presence of clinical neglect were similar (S1 Fig).

**Table 4:** Univariate and multivariate analyses of factors predicting clinical neglect.

	Univariate analysis		Multivariate analysis	
	OR (95% CI)	P	OR (95% CI)	P
**Age**	1.07 (1.02–1.13)	0.01	–	
**Sex**				
Male	1			
Female	1.50 (0.48–4.70)	0.486	–	
**Lesions**				
Rt. hemisphere	1			
Lt. hemisphere	0.04 (0.00–0.29)	<0.001	**–**	
Controls	0.04 (0.00–0.29)	<0.001	**–**	
**CTST**				
Horizontal deviation	1.21 (1.07–1.35)	0.002	1.17 (1.01–1.36)	0.037
Selection tendency	3.60 (1.84–7.04)	<0.001	2.57 (1.34–4.94)	0.005
Elapsed time	1.04 (1.01–1.07)	0.004	–	
**Conventional tests**				
Line bisection	3.66 (1.63–8.20)	0.002	–	
Star cancellation	2.45 (0.54–11.17)	0.246	–	
Total neglect score	2.42 (1.67–5.04)	0.018	–	

CTST = the computerized table setting test.

## Discussion

This study demonstrated that the CTST was feasible and comparable with conventional neglect tests. The CTST can simultaneously measure three parameters, including the horizontal deviation from the midline, the selection tendency for right-side dishes, and the elapsed time to complete the test. As soon as the subjects set the table with all the dishes, the CTST instantly provides the scores on the three parameters as well as the probability of neglect. Although the conventional paper-and-pen based neglect tests are useful [3–5], these tests require considerable time to perform and interpret the data compared with the CTST [21]. A stroke is prevalent in older individuals and patients with neglect tend to have a decreased attention span. In addition, approximately 20–40% of patients with RHI have anosognosia [22], which may prevent the detection of neglect [23]. Therefore, possible sources of misunderstanding, errors, and considerable time in performing complex paper-and-pen based tests exist.

Correlation analysis revealed that all three parameters of the CTST were significantly correlated with the total neglect score. Comparison between groups showed that the CTST may provide information about different aspects of neglect phenomenon. The horizontal deviation of the CTST can differentiate patients with neglect from others like the conventional tests. Using the horizontal deviation or the conventional tests, there was no significant difference between RHI without neglect group and other groups. Although the selection tendency and the elapsed time were not different between RHI with neglect group and RHI without neglect group, the selection tendency to right side was increased in the patients with RHI whether they had clinical neglect or not. The selection tendency may uncover subclinical neglect in patients with RHI. Subclinical neglect is known to reflect inattention from right hemispheric lesions. A subtle rightward orienting bias due to inattention cannot be detected by bedside neurological examinations or conventional neglect tests [24,25]. Because subtle clinical neglect can adversely affect the ability to complete complex daily activities during stroke recovery, detecting subclinical neglect may be important in stroke rehabilitation [12,24]. The overall sensitivity of the CTST were highest followed by the star cancellation test, the selection tendency of the CTST, the line bisection test, the elapsed time, and the horizontal deviation of the CTST. Low sensitivity of the horizontal deviation is probably related to the short horizontal length of a tablet device [26]. Among the parameters of the CTST, the horizontal deviation and elapsed time significantly correlated with the infarction volume [13,27]. Given that higher parameter values represent a higher probability of the presence of neglect, these features are in line with previous studies that showed the presence of neglect was associated with a larger infarction volume [13,27].

Various tests have been developed to detect and characterise neglect [3–5]. The line bisection test and the star cancellation test are commonly used as screening neglect tests. However, they tend to underestimate the presence of neglect and are not adequate to assess the presence of neglect in everyday life. The line bisection test has a considerable false positive rate for hemianopic patients without neglect syndrome and low test-retest reliability [28–30]. It has been known that the word reading [21] and the double cancellation tasks [31] are more sensitive than the paper-and-pencil tests. In addition, the behavioral assessments are more sensitive to detect the clinically significant neglect in everyday life. Among the behavioral assessment, the Catherine Bergego Scale (CBS) has been known as a reliable, valid and sensitive test, which can easily be performed during rehabilitation [21]. Comparison with a more sensitive neglect test or a test reflecting the activities of daily living (ADLs) might be helpful to confirm the usefulness of the CTST. However, we included patients with the ischemic stroke at acute stage and our patients had severe neurological deficits (NIHSS score 7.5 ± 4.3). These patients also required special care in the stroke unit. Moreover, about half of study patients (9 out of 20) had anosognosia that made more difficult to perform the complex tests.

Because neglect is multi-modal with heterogeneous features, a single test cannot completely evaluate neglect syndrome. For that reason, a battery of tests is more sensitive than a single neglect test, and recommended [3,5,21]. A battery of tests requires considerable time, and some tests cannot be easily replicated [21]. Therefore, there remains a need for a single test which is quick, easy to perform, reproducible, and reasonably sensitive [32]. The baking tray test is similar to the CTST. In the baking tray test, a subject is instructed to spread out 16 cubes as evenly as possible as if they were buns on a baking tray. When the task is finished, the presence of neglect is determined by the number of cubes in each half-field [32]. Like the baking tray test, the CTST can uncover spatial neglect, motor neglect, and inattention after stroke. In addition, the CTST has features that are absent from the baking tray test, including calculation of results in real-time, the presenting the probability of the presence of neglect, and overcomes cultural differences. Because table setting is a popular task across cultures, a neglect patient can easily understand and perform the CTST. The number of physicians who are familiar with use of mobile devices in daily clinical practice is increasing [33,34] and many studies demonstrated that mobile applications are quick and accurate for measuring neurological deficit and clinical diagnosis [8,9,35]. The CTST provides the probability of the presence of clinical neglect as soon as the test is completed. Even a physician who is not trained or familiar with neglect tests can instantly obtain the probability of neglect. Therefore, the physicians who do not specialise in neurology can easily identify neglect patients using the CTST, and refer the patients.

Time in the clinical setting is always limited especially in acute period after stroke. The CTST was developed for screening neglect in acute stroke and the CTST was performed using a portable tablet device. In this regard, the CTST has potential benefits. First, the conventional paper-and-pencil tests could not be performed because of a difficulty in cooperation. Indeed, during the star cancellation test, two study patients could not undergo the test due to a poor cooperation, whereas all patients complete the CTST. Second, time required to perform the CTST was shorter than the conventional tests. Mean elapsed time of the CTST was only 74.75 ± 52.41 sec, whereas it had been known that elapsed time of the conventional neglect tests are up to several minutes. Third, there exists additional considerable time measuring and calculating the results of the paper-and-pencil tests. For example, the line bisection test requires measuring the deviation with a ruler and the star cancellation test requires counting omitted items. Those tests also require comparing with normative data. In contrast, the CTST instantly provides the scores of three parameters and the probability of neglect as soon as the subjects set the table with all dishes. Fourth, some elderly patients have a difficulty in understanding complex tasks. Because table setting is a popular task across cultures, they can understand and perform the CTST without sizable difficulty. In addition, examiner may explain the CTST in a very short time. Fifth, The CTST revealed subtle neglect in patients with RHI. Detecting subclinical neglect is important in stroke rehabilitation [24,25]. Lastly, we performed the follow-up tests in patients who had RHI with clinical neglect to show usability of the CTST. The horizontal deviation and the selection tendency of the CTST were significantly improved during follow-up. These results suggested that the CTST might be a useful tool to quantify improvement in patients with neglect.

Our study has some limitations. First, the CTST is a screening test to detect the presence of neglect in acute stage. We have to address that the CTST cannot replace neglect tests assessing ADLs that had been known to be useful in planning rehabilitation for the patients with neglect. For that purpose, more comprehensive neglect tests such as CBS might be helpful [36]. Second, we compared the CTST with two popular paper-and-pencil tests of the line bisection and the star cancellation test. The sensitivity of those tests might be lower than more sensitive tests. Unfortunately, we do not have a local version of more sensitive tests such as the word reading [21] and the double cancellation tasks [31] and their normative data. We could not perform those tests. In addition, comparison with a battery of neglect test might be helpful to confirm the usefulness of the CTST. Third, although we made an equation to predict the probability of clinical neglect, further studies that apply the CTST to other cohorts is needed. Lastly, a larger sample size with longitudinal assessment would be needed to uncover extra benefits to address whether the CTST can predict outcome in chronic stage after stroke.

## Conclusions

In conclusion, we found that the CTST was feasible and the results were comparable to conventional neglect tests. This new application may be useful for the initial diagnosis and follow-up of neglect patients.

## Supporting Information

S1 Fig Nomogram to predict the presence of clinical neglect in patients with acute ischemic stroke. We constructed a nomogram according to these two parameters. In the nomogram, each point of the horizontal deviation and selection tendency was calculated. A sum of each point was regarded as a total point. A corresponding probability of clinical neglect can be estimated from each total point. For example, if the horizontal deviation is 15 mm and the selection tendency is 4, the points of each parameter are 35 and 17, respectively; therefore the total point is 52. The total point of 52 was translated into a probability of neglect of 77.8% (A). An internal validation of the nomogram revealed that the ability of the nomogram to discriminate the presence of clinical neglect was excellent [area under the curve (AUC) of 0.937 (95% CI, 0.873 to 1.000)] (B). In terms of calibration, the predicted probabilities from the equation and the actual probability of the presence of clinical neglect were similar (C).
(PNG)

S1 Table Post-hoc analysis to compare the results of the CTST and conventional tests across groups. (DOCX)

S2 Table Comparison of infarction volume in patients with RHI with and without neglect. (DOCX)
