# Supplementary figures and images for: The Computerized Table Setting Test for Detecting Unilateral Neglect
Source: PLoS One. 2016 Jan 15;11(1):e0147030. doi: 10.1371/journal.pone.0147030 (PMC4714760; doi:10.1371/journal.pone.0147030)

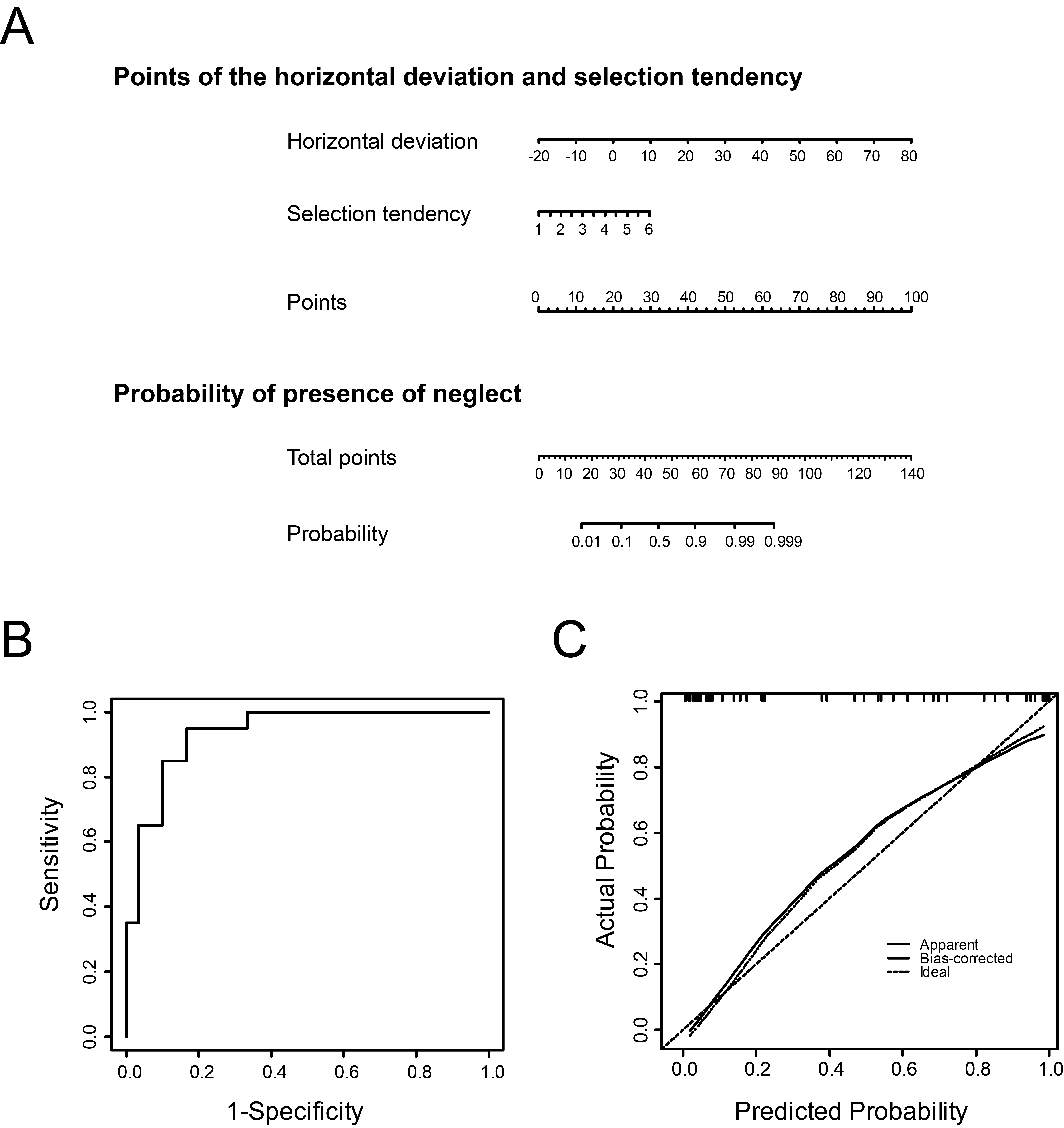

Supplement: S1 Fig — We constructed a nomogram according to these two parameters. In the nomogram, each point of the horizontal deviation and selection tendency was calculated. A sum of each point was regarded as a total point. A corresponding probability of clinical neglect can be estimated from each total point. For example, if the horizontal deviation is 15 mm and the selection tendency is 4, the points of each parameter are 35 and 17, respectively; therefore the total point is 52. The total point of 52 was translated into a probability of neglect of 77.8% (A). An internal validation of the nomogram revealed that the ability of the nomogram to discriminate the presence of clinical neglect was excellent [area under the curve (AUC) of 0.937 (95% CI, 0.873 to 1.000)] (B). In terms of calibration, the predicted probabilities from the equation and the actual probability of the presence of clinical neglect were similar (C). (PNG) [file pone.0147030.s001.png]
