# Supplementary material for: The Computerized Table Setting Test for Detecting Unilateral Neglect
Source: PLoS One. 2016 Jan 15;11(1):e0147030. doi: 10.1371/journal.pone.0147030 (PMC4714760; doi:10.1371/journal.pone.0147030)
Supplement: S1 Table — (DOCX) [file pone.0147030.s002.docx]

# S1 Table. Post-hoc analysis to compare the results of the CTST and conventional tests across groups.

|  | RHI with neglect vs. RHI without neglect | RHI with neglect vs. LHI | RHI with neglect vs. Control | RHI without neglect vs. LHI | RHI without neglect vs. Control | LHI vs. Control |
| --- | --- | --- | --- | --- | --- | --- |
| **CTST** |  |  |  |  |  |  |
| Horizontal deviation | 0.004 | 0.001 | 0.003 | 0.491 | 0.152 | 0.350 |
| Selection tendency | 0.090 | < 0.001 | < 0.001 | 0.005 | 0.007 | 0.891 |
| Elapsed time | 0.105 | 0.031 | 0.001 | 0.342 | 0.085 | 0.325 |
|  |  |  |  |  |  |  |
| **Conventional test** |  |  |  |  |  |  |
| Line bisection | 0.034 | 0.003 | 0.001 | 0.186 | 0.383 | 0.360 |
| Star cancellation | 0.002 | 0.002 | 0.002 | 0.721 | 0.353 | 0.324 |
| Total neglect score | 0.007 | 0.001 | 0.002 | 0.322 | 0.510 | 0.742 |

The values are expressed as the p-value using a Bonferroni correction for multiple comparisons. CTST = the computerised table setting test; RHI = right hemispheric infarction; LHI = left hemispheric infarction; NIHSS, National Institutes of Health Stroke Scale.
