# Supplementary material for: The Computerized Table Setting Test for Detecting Unilateral Neglect
Source: PLoS One. 2016 Jan 15;11(1):e0147030. doi: 10.1371/journal.pone.0147030 (PMC4714760; doi:10.1371/journal.pone.0147030)
Supplement: S2 Table — (DOCX) [file pone.0147030.s003.docx]

**S2 Table. Comparison of infarction volume in patients with RHI with and without neglect.**

| Patient | Location | Volume | NIHSS | Visual | Auditory | Tactile | Anosognosia | Asomatognosia |
| --- | --- | --- | --- | --- | --- | --- | --- | --- |
|  |  |  |  |  |  |  |  |  |
| **RHI with neglect** | |  |  |  |  |  |  |  |
| 1 | F-P-T | 17.74 | 13 | - | + | + | - | - |
| 2 | F-T | 15.26 | 3 | - | - | + | - | - |
| 3 | F-T | 65.94 | 11 | - | - | + | + | + |
| 4 | F-P-T | 4.93 | 4 | + | + | + | + | - |
| 5 | P-T | 0.58 | 4 | + | + | + | + | - |
| 6 | F-P-T | 63.83 | 11 | - | + | + | + | + |
| 7 | F-P-T | 22.68 | 2 | + | + | - | - | - |
| 8 | F-T | 24.55 | 12 | - | + | + | + | + |
| 9 | F-P-T | 9.96 | 5 | + | + | + | - | - |
| 10 | F-P-T | 25.67 | 2 | - | + | - | - | - |
| 11 | P-T | 74.78 | 4 | - | + | + | - | - |
| 12 | F-P-T | 14.67 | 17 | + | + | + | - | - |
| 13 | F-P-T | 39.98 | 11 | + | + | + | + | + |
| 14 | F-P-T | 19.91 | 5 | - | - | + | - | - |
| 15 | F-P-T | 23.26 | 13 | - | + | + | - | + |
| 16 | F-T | 104.08 | 12 | + | - | + | + | + |
| 17 | F-P-T | 178.19 | 6 | - | - | - | + | - |
| 18 | F-P-T | 47.97 | 4 | + | - | - | - | - |
| 19 | F-T | 28.61 | 4 | - | - | + | + | - |
| 20 | P-T | 32.73 | 6 | + | - | + | - | - |
| Mean |  | 40.77 ± 41.52 |  |  |  |  |  |  |
|  | |  |  |  |  |  |  |  |
| **RHI without neglect** | | |  |  |  |  |  |  |
| 21 | F-P | 0.59 | 4 | - | - | - | - | - |
| 22 | P-T | 45.75 | 0 | - | - | - | - | - |
| 23 | F-T | 12.09 | 1 | - | - | - | - | - |
| 24 | F-P-T | 14.40 | 1 | - | - | - | - | - |
| 25 | F-P-O | 4.66 | 2 | - | - | - | - | - |
| 26 | F-P-T | 1.13 | 1 | - | - | - | - | - |
| 27 | F-P-T | 6.89 | 2 | - | - | - | - | - |
| 28 | P | 3.35 | 2 | - | - | - | - | - |
| 29 | P | 0.45 | 0 | - | - | - | - | - |
| 30 | F | 4.94 | 1 | - | - | - | - | - |
| Mean |  | 9.43 ± 13.60 |  |  |  |  |  |  |

RHI = right hemispheric infarction; F = the frontal lobe; P = the parietal lobe; T = the temporal lobe; O = the occipital lobe.
